# Some Preachers and Subjects for Hospital Sunday

**Published:** 1889-06-22

**Authors:** 


### Some Preachers and Subjects on Hospital Sunday, 1889.

We have received the following announcements, but unusual pressure on our space this week renders it impossible to give a full list as we wished to do:

Bayswater Synagogue.—On Saturday last, the 15th inst., the Rev. Dr. Hermann Adler, Delegate Chief Rabbi, preached on "The Culture of the Heart," and referred to one aspect of Hospital Sunday as contributing to develop sympathy. On the 22nd inst., which is observed as Hospital Sabbath by the Jewish community, he will preach on "The Teachings of Sickness," taking as his text Deuteronomy 32, xxxix., "I wound and I heal."

Claverton-street Wesleyan Chapel.—Subject: "Christian Duty as interpreted for us by our Lord's Example." "He took our Infirmities and bore our Sickness." In the City of London there is real, intense, extensive suffering. To many we can give complete relief. To some, alleviation; to all, sympathy, prayer, and proclamation of the old, old story of a Saviour's love. Christianity cares for the bodies of men as well as their souls. Preacher, Rev. R. Bentley.

Homerton, N.E.—The Rev. W. Baker, B.D.; subject, "Blind Bartimeas," Mark x., 46-52, "Jesus the Sight-Giver to the Needy," morning. Evening, Rev. J. R. Gilpin, Acts iv., 36, 37; subject, "The Son of Consolation."

Islington Presbyterian Church.—11 a.m. Subject, "Selfishness Uprooted by the Knowledge of the Character of God," Rev. Thain Davidson, DD.

Methodist Free Church, Bellenden-road, Peckham, S.E.—Rev. R. Wilton. Subjects: Morning, "The Thorn in the Flesh;" evening, "The Good Samaritan."

North Finchley Congregational Church.—Rev. Thos. Hill. Morning at 11 o'clock, "The Twofold Ministry of Christianity;" evening at 6.30, "The Capernium Miracles."

St. Mary, Newington.—Morning, Rev. F. Fisher; evening, Rev. George Palmer, on "The Gifts of Healing," 1 Cor. xii., 30.

St. Matthew's Church, Oakley-square, N.W.—Preachers: 11 a.m., the Vicar (the Rev. H. E. B. Arnold, M.A.), "The Reciprocal Duties of Hospitals towards the Churches in the Matters of Centralisation and Economy"; 3.15 p.m., the Vicar, Children's Flower Service; 7 p.m., the Rev. J. C. Berkeley, B.A., senior curate, the church of Watford, Herts, "The Hospitals and the Poor."

St. Stephen's, Hounslow.—11 a.m., Rev. Henry Layton, vicar; at 6.30 p.m., Rev. Augustus E. Farrar, curate.

